# Residue 208 of UGT1A7 Determines the Functional Consequences of CYP3A4-UGT1A7 Association in Living Cells

**DOI:** 10.3390/biology15201895

**Published:** 2026-10-21

**Authors:** Natsuki Egoshi, Yuu Miyauchi, Shin’ichi Ikushiro, Kiyoshi Nagata, Yasushi Yamazoe, Peter I. Mackenzie, Hideyuki Yamada, Yuji Ishii

**Affiliations:** 1 Laboratory of Molecular Life Sciences, Graduate School of Pharmaceutical Sciences, Kyushu University, Fukuoka 812-8582, Japanyuu.miyauchi.b6@tohoku.ac.jp (Y.M.);; 2 Division of Pharmaceutical Cell Biology, Graduate School of Pharmaceutical Sciences, Kyushu University, Fukuoka 812-8582, Japan; 3 Laboratory of Pharmacogenomics, Graduate School of Pharmaceutical Sciences, Tohoku University, Sendai 980-8578, Japan; 4 Biotechnology Research Center, Faculty of Engineering, Toyama Prefectural University, Imizu 939-0398, Japan; ikushiro@pu-toyama.ac.jp; 5 Departments of Environmental Health, Faculty of Pharmaceutical Sciences, Tohoku Medical and Pharmaceutical University, Sendai 981-8558, Japan; nagataki@mac.com; 6 Division of Drug Metabolism and Molecular Toxicology, Graduate School of Pharmaceutical Sciences, Tohoku University, Sendai 980-8578, Japan; yamazoe@m.tohoku.ac.jp; 7 Department of Clinical Pharmacology, College of Medicine and Public Health, Flinders University, Adelaide, SA 5042, Australia; peter.mackenzie@flinders.edu.au

**Keywords:** CYP3A4, UGT1A7, protein–protein interaction, FRET, glucuronidation, pharmacogenetics, UDP-glucuronosyltransferase, Cytochrome P450

## Abstract

Cytochrome P450 3A4 (CYP3A4) and UDP-glucuronosyltransferase 1A7 (UGT1A7) are known to interact functionally on the endoplasmic reticulum membrane. In a previous study, CYP3A4 enhanced the activities of UGT1A7*1 and UGT1A7*2 but suppressed that of UGT1A7*3, suggesting a role for residue 208 in CYP3A4-dependent regulation. To test this hypothesis, we investigated UGT1A7*4, a naturally occurring variant carrying the W208R substitution. Enzyme kinetic analyses demonstrated that UGT1A7*4 retained basal catalytic activity comparable with that of wild-type UGT1A7*1. However, co-expression of CYP3A4 suppressed UGT1A7*4 activity, whereas it enhanced UGT1A7*1 activity. This opposite regulatory response was consistently observed with 4-methylumbelliferone (4-MU), SN-38, and 4-hydroxybiphenyl (4-OHBP). Pull-down assays showed that UGT1A7*1, UGT1A7*4, and a W208A mutant all retained detectable association with CYP3A4. FRET analysis further demonstrated direct interaction between CYP3A4 and both UGT1A7 variants in living cells. These findings indicate that residue 208 influences the functional consequences of CYP3A4-mediated regulation without abolishing physical association between CYP3A4 and UGT1A7. We propose that residue 208 may function as a determinant, and potentially a molecular switch, governing the outcome of CYP3A4-dependent modulation of UGT1A7 activity.

## Simple Summary

Cytochrome P450 3A4 (CYP3A4) and UDP-glucuronosyltransferase 1A7 (UGT1A7) are drug-metabolizing enzymes that interact physically and functionally on the endoplasmic reticulum membrane. In this study, we investigated how a naturally occurring amino acid substitution at residue 208 affects this interaction. We found that the W208R substitution did not disrupt detectable association between CYP3A4 and UGT1A7 but reversed the functional effect of CYP3A4 from activation to suppression of UGT1A7 activity. Furthermore, we demonstrated CYP3A4–UGT1A7 interaction in living cells using fluorescence resonance energy transfer (FRET). These findings suggest that residue 208 may function as a molecular switch that determines the functional consequences of CYP3A4–UGT1A7 interaction and provide new insights into regulatory mechanisms among drug-metabolizing enzymes.

## 1. Introduction

UDP-glucuronosyltransferases (UGTs) are major phase II drug-metabolizing enzymes that catalyze the conjugation of glucuronic acid to a wide variety of endogenous and exogenous compounds, thereby facilitating their elimination from the body [1]. Together with cytochrome P450 (CYP, P450) enzymes, UGTs play a central role in the biotransformation and detoxification of xenobiotics [1,2,3]. While CYP enzymes catalyze oxidative reactions in phase I metabolism [3], UGTs generally function in subsequent conjugation reactions although drugs such as non-steroidal anti-inflammatory drugs (NSAIDs) are directly glucuronidated without undergoing significant phase I reactions [4,5]. Consequently, functional variability in either enzyme family can significantly influence drug efficacy, toxicity, and interindividual variation in pharmacokinetics.

Although CYPs and UGTs were traditionally regarded as independent enzyme systems, accumulating evidence has revealed extensive protein–protein interactions among drug-metabolizing enzymes within the endoplasmic reticulum membrane [6,7,8,9,10,11,12]. Previous studies have demonstrated physical and functional interactions between CYP3A4 and several UGT isoforms, including UGT2B7, UGT1A1, UGT1A6, UGT1A9, and UGT1A7 [7,8,9,10,11,12]. These interactions are capable of altering catalytic activity, substrate selectivity, and kinetic parameters, indicating that the protein–protein association represents an important regulatory mechanism for drug metabolism.

Interactions among drug-metabolizing enzymes are now recognized as important determinants of metabolic function. Initial studies focused on interactions among distinct cytochrome P450 species, which were shown to alter catalytic behavior through formation of heteromeric complexes [13,14,15,16,17,18,19].

UGT1A7 is primarily expressed in extrahepatic tissues, particularly in the gastrointestinal tract, and contributes to the glucuronidation of numerous xenobiotics, including the active irinotecan metabolite SN-38 and metabolites of environmental carcinogens [20,21,22]. Several genetic polymorphisms have been identified within the UGT1A7 gene, and some variants have been associated with altered drug metabolism and drug susceptibility [20]. In particular, UGT1A7*1 represents the reference sequence, whereas UGT1A7*3 contains three amino acid substitutions (N129K, R131K, and W208R) [22]. UGT1A7*3 has attracted considerable interest because of its altered responses to CYP3A4-dependent regulation [10].

In our previous study, co-expression of CYP3A4 enhanced the glucuronidation activities of UGT1A7*1 and UGT1A7*2 but suppressed that of UGT1A7*3 [10]. Although UGT1A7*3 had previously been regarded as a low-activity variant [20,22], we found that its basal catalytic activity and kinetic properties were largely comparable with those of wild-type UGT1A7*1 [10]. These findings suggested that UGT1A7 polymorphisms primarily affect susceptibility to CYP3A4-mediated regulation rather than intrinsic catalytic activity. Comparison of UGT1A7 variants further indicated that the most distinctive difference between the CYP3A4-responsive variants (*1 and *2) and the CYP3A4-suppressed variant (*3) was the W208R substitution, suggesting that residue 208 plays a critical role in determining the functional consequences of CYP3A4–UGT1A7 interactions. However, direct experimental evidence supporting this hypothesis has been lacking.

To address this issue, we focused on UGT1A7*4, a naturally occurring variant that contains the W208R substitution in the absence of the N129K and R131K substitutions found in UGT1A7*3. By comparing UGT1A7*4 with wild-type UGT1A7*1, we sought to determine whether residue 208 is responsible for the differential regulation of UGT1A7 by CYP3A4. In addition, we generated a W208A mutant to further evaluate the structural and functional significance of this residue. Enzyme kinetic analyses were performed using multiple UGT1A7 substrates, including 4-methylumbelliferone (4-MU), SN-38, and 4-hydroxybiphenyl (4-OHBP). To determine whether altered CYP3A4 regulation reflects changes in the protein–protein association, we examined interactions between CYP3A4 and UGT1A7 variants using complementary biochemical and cellular approaches, including pull-down assays and fluorescence resonance energy transfer (FRET) analysis.

## 2. Materials and Methods

### 2.1. Chemicals and Reagents

4-Methylumbelliferone (4-MU) and alamethicin were purchased from Sigma-Aldrich (St. Louis, MO, USA). UDPGA and 4-MU glucuronide were purchased from Nacalai Tesque Inc. (Kyoto, Japan). 4-Hydroxybiphenyl (4-OHBP) was purchased from FUJIFILM Wako Pure Chemical Corporation (Osaka, Japan). SN-38 and SN-38 glucuronide were kindly provided by Yakult Honsha Co., Ltd. (Tokyo, Japan). 4-OHBP glucuronide was isolated by the method of Dodgson et al. [23] from the urine of rabbits administered with 4-OHBP [24]. All other chemicals were of analytical grade or the highest quality commercially available.

### 2.2. Construction of UGT1A7 Variants

Human UGT1A7*1 cDNA previously constructed in our laboratory [10] was used as the wild-type construct. UGT1A7*4 carrying the W208R substitution was generated by site-directed mutagenesis using UGT1A7*1 as a template. A UGT1A7 W208A mutant was similarly generated by PCR-based mutagenesis. All constructs were verified by DNA sequencing.

Primer sequences and PCR conditions are summarized in Supplementary Tables S1 and S2 and in Supplemental Methods S1.

### 2.3. Expression of UGT1A7 Variants and CYP3A4 in Sf9 Cells

Recombinant baculoviruses were prepared using the Bac-to-Bac Baculovirus Expression System (Thermo Fisher Scientific, Waltham, MA, USA). Sf9 cells were maintained in serum-supplemented Sf-900 II medium and infected with recombinant baculoviruses expressing UGT1A7 variants and/or CYP3A4 as described previously [10].

For CYP3A4-containing cultures, hemin was added 24 h after infection to facilitate formation of catalytically active holo-CYP3A4. Cells were harvested approximately 60 h after infection, and microsomes were prepared by differential centrifugation.

Multiplicities of infection (MOIs) were optimized to obtain comparable UGT1A7 expression levels and are summarized in Supplementary Table S3.

### 2.4. Immunoblot Analysis

Microsomal proteins were separated by SDS-PAGE and transferred onto polyvinylidene difluoride membranes. UGT1A7 proteins were detected using a rabbit anti-UGT1A antibody [25], whereas CYP3A4 was detected using an anti-CYP3A antibody (monoclonal antibody-CYP3A, WB-MAB-3A, Corning Gentest™ (Woburn, MA, USA)). Following incubation with horseradish peroxidase-conjugated secondary antibodies, immunoreactive bands were visualized by enhanced chemiluminescence using a ChemiDoc imaging system (Bio-Rad Laboratories, Hercules, CA, USA).

### 2.5. Enzyme Activity Assays

#### 2.5.1. 4-Methylumbelliferone Glucuronidation

UGT activity toward 4-MU was determined using microsomal preparations as described previously [26]. Reaction mixtures contained potassium phosphate buffer (pH 7.4), MgCl_2_, alamethicin, UDPGA, substrate, and microsomal protein in 0.3 mL. After incubation at 37 °C, reactions were terminated by addition of 0.1 mL 1 M trichloroacetic acid, and glucuronide formation was quantified by HPLC equipped with a fluorescent detector.

#### 2.5.2. SN-38 Glucuronidation

SN-38 glucuronidation activity was determined under conditions similar to those described above [27]. Quantification of SN-38 glucuronide was performed by HPLC with fluorescence detection.

#### 2.5.3. 4-Hydroxybiphenyl Glucuronidation

UGT activity toward 4-OHBP was determined under conditions similar to those described above for 4-MU. 4-OHBP glucuronide was quantified by HPLC with a fluorescence detection condition similar to those described above.

#### 2.5.4. UDPGA-Dependent Kinetic Analysis

To evaluate cofactor utilization, UDPGA concentrations were varied while maintaining a fixed concentration of 4-MU. Apparent kinetic parameters for UDPGA were determined by nonlinear regression.

Detailed chromatographic conditions are summarized in Supplementary Table S4.

### 2.6. Pull-Down Assay

Protein–protein interactions between CYP3A4 and UGT1A7 variants were examined using a His-tag pull-down assay method as described previously [11].

UGT1A7*1, UGT1A7*4, and W208A were fused to a C-terminal hemagglutinin (HA) epitope tag and transiently expressed in COS-1 cells in the presence or absence of C-terminally His-tagged CYP3A4 (CYP3A4-His). Forty-eight hours after transfection, cell lysates were prepared and incubated with Ni^2+^-NTA agarose.

After extensive washing, bound proteins were eluted with imidazole-containing buffer and analyzed by immunoblotting using anti-HA and anti-His antibodies.

### 2.7. Construction of Fluorescent Fusion Proteins

For fluorescence resonance energy transfer (FRET) analysis, UGT1A7*1 and UGT1A7*4 were fused to AcGFP1-N1 (Clontech Laboratories, Mountain View, CA, USA) at their C-termini. Human calnexin (CNX) fused to AcGFP1 was used as a control protein. CYP3A4 was fused to mCherry (Clontech Laboratories) at its C-terminus.

All fusion constructs were verified by DNA sequencing prior to use.

### 2.8. FRET Analysis

COS-1 cells were co-transfected with AcGFP1- and mCherry-tagged expression vectors and incubated for 48 h.

Live-cell imaging was performed using a laser-scanning confocal microscope. AcGFP1 fluorescence was detected at 490–550 nm after excitation at 488 nm, whereas mCherry fluorescence was detected at 560–700 nm after excitation at 555 nm. FRET signals were obtained by exciting AcGFP1 and monitoring fluorescence emission within the mCherry detection range.

We then processed the images using the following equation to generate images representing FRET efficiency as luminance. The constants CoA and CoB were calculated using samples expressing only mCherry and samples expressing only AcGFP1, respectively, to determine the proportion of mCherry fluorescence that leaks into the FRET channel due to factors other than the FRET phenomenon and the proportion of AcGFP1 fluorescence that leaks into the mCherry fluorescence detection filter. Since these values are specific to the combination of microscope and vector used, the same values were adopted for all FRET analyses in this study (CoA = 0.097, CoB = 0.032).

FRET_Corr_ = FRET_Raw_ − CoA mCherry − CoB AcGFP1

CoA: The percentage of mCherry excitation crosstalk in the FRET image calculated from the control mCherry sample.

CoB: The percentage of AcGFP1 emission crosstalk in the FRET channel calculated from the control AcGFP1 sample.

$$\mathrm{Ratio}\;(\mathrm{FRET}\;\mathrm{efficiency})=({\mathrm{FRET}}_{\mathrm{Corr}}/\mathrm{AcGFP}1)\;\times \;100=\left[\frac{\frac{{\mathrm{FRET}}_{\mathrm{Corr}}}{\mathrm{Imax}\left(\mathrm{FRETcorr}\right)}}{\frac{\mathrm{AcGFP}1}{\mathrm{Imax}\left(\mathrm{AcGFP}1\right)}}\right]\times 100$$

Imax: The maximum gray value.

FRET efficiency images were generated using sensitized emission analysis after correction for spectral bleed-through. Ten cells were analyzed for each experimental condition.

### 2.9. Data Analysis

Enzyme kinetic parameters were estimated by nonlinear regression using the Michaelis–Menten equation:

$$V=\frac{V_{\max}\left[S\right]}{K_{m}+\left[S\right]}$$

Curve fitting and statistical analyses were performed using GraphPad Prism software ver. 8 (GraphPad Software, La Jolla, CA, USA). Data are presented as mean ± S.E. Statistical significance was assessed using the tests indicated in the respective figure legends. Differences were considered statistically significant when *p* < 0.05.

## 3. Results

### 3.1. CYP3A4 Differentially Modulates UGT1A7*1 and UGT1A7*4

**Figure 1:**
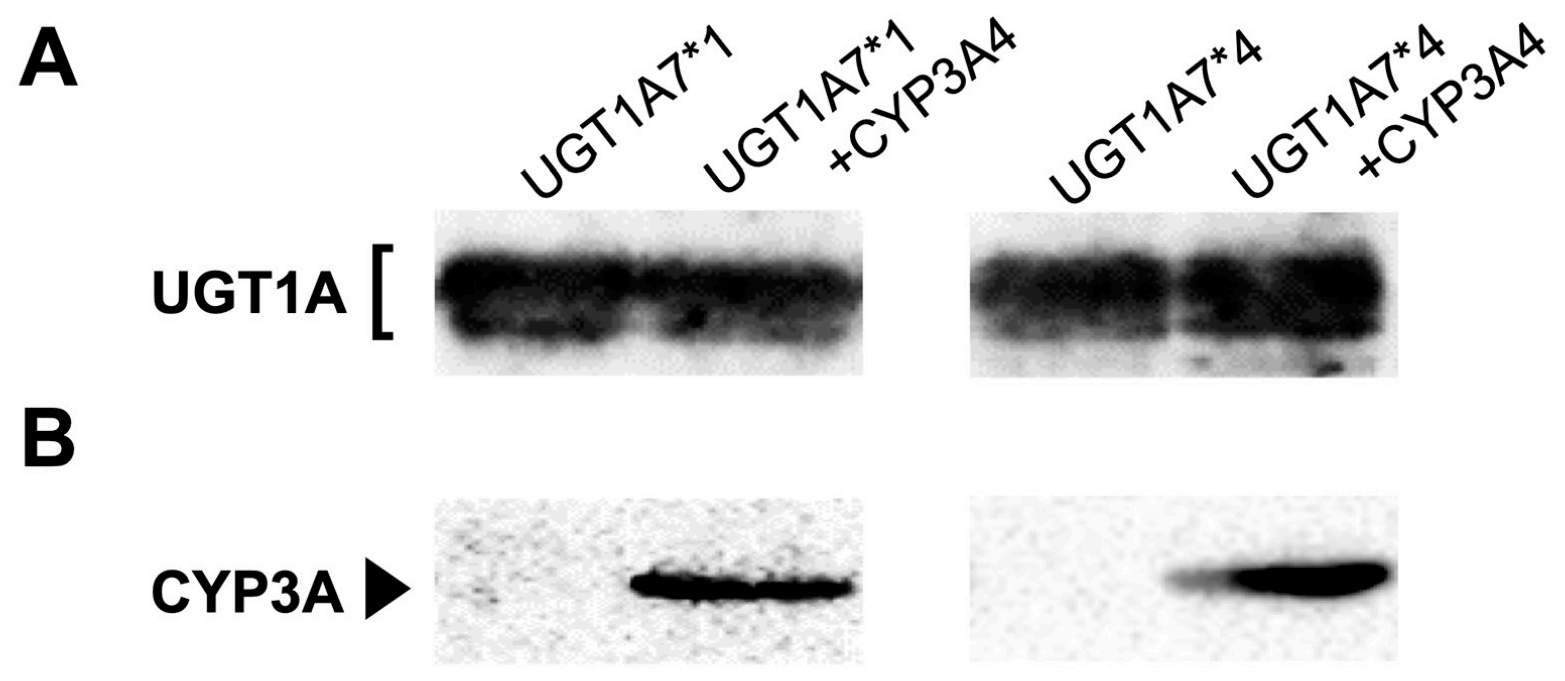
Expression of UGT1A7*1 and UGT1A7*4 in the presence or absence of co-expressed CYP3A4. In (**A**), Western blotting was performed using a rabbit anti-rat UGT1A common antibody [23] as the primary antibody. In (**B**), Western blotting was performed using a mouse anti-human CYP3A antibody (WB-MAB-3A: BD Gentest) as the primary antibody. All lanes represent the same samples as those in Panel (**A**).

To determine whether the W208R substitution influences CYP3A4-mediated regulation of UGT1A7, we prepared microsomes expressing either UGT1A7*1 or UGT1A7*4 in the presence or absence of CYP3A4 (Figure 1) and compared their glucuronidation activities using multiple substrates.

**Figure 2:**
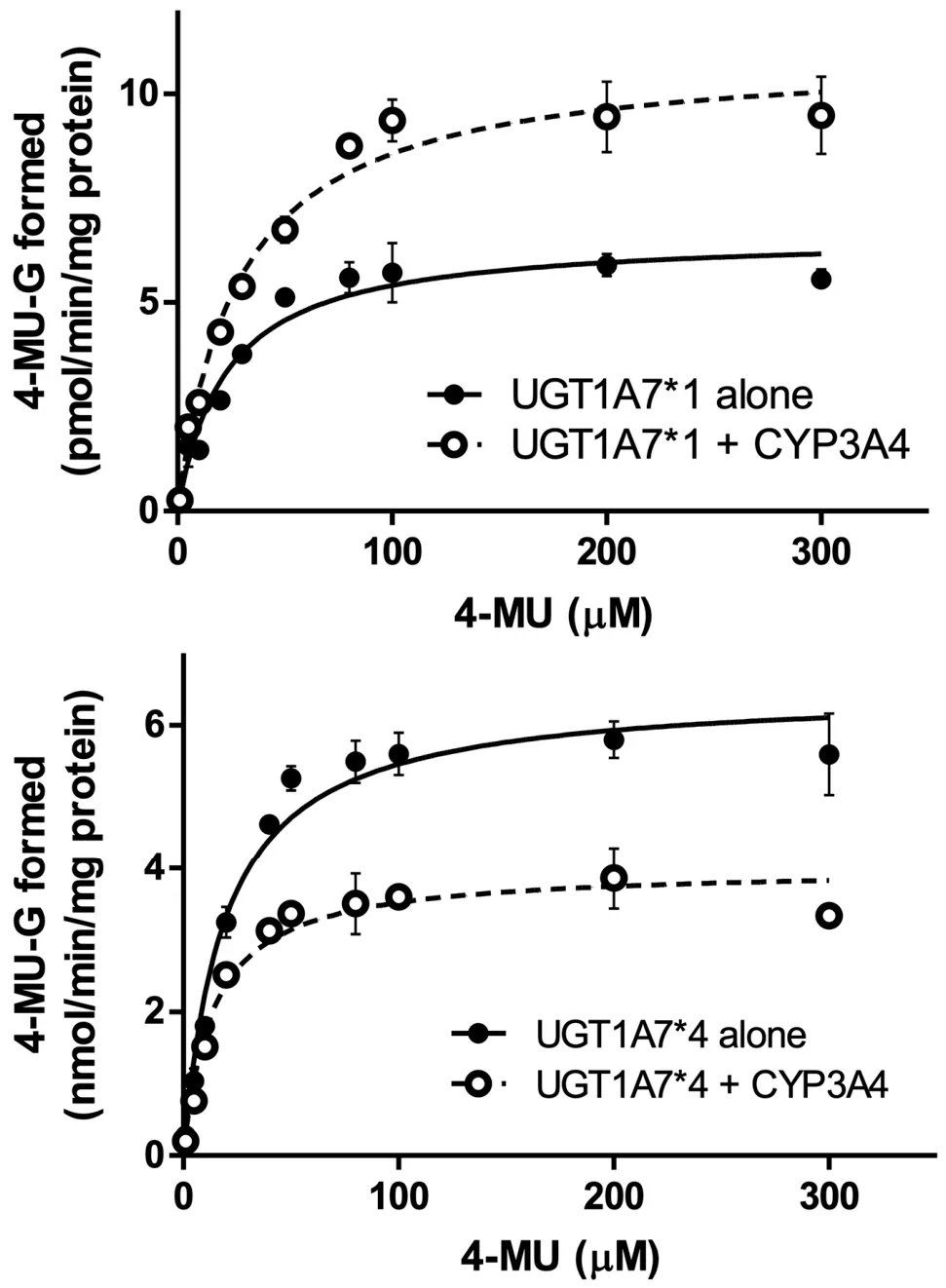
Differential effects of CYP3A4 on 4-MU glucuronidation catalyzed by UGT1A7*1 and UGT1A7*4. (**Upper panel**) Michaelis–Menten plots for 4-MU glucuronidation catalyzed by UGT1A7*1 in the absence and presence of CYP3A4. (**Lower panel**) Michaelis–Menten plots for 4-MU glucuronidation catalyzed by UGT1A7*4 in the absence and presence of CYP3A4.

The effect of CYP3A4 on UGT1A7 activity was first examined using 4-MU as a substrate. Kinetic analyses demonstrated that co-expression of CYP3A4 significantly enhanced UGT1A7*1-catalyzed 4-MU glucuronidation, resulting in a marked increase in the apparent *V*max value with little change in substrate affinity (Figure 2, Table 1). In contrast, CYP3A4 co-expression significantly decreased the *V*max of UGT1A7*4-mediated glucuronidation (Figure 2, Table 1). The apparent *K*m values were only minimally affected by CYP3A4 in either variant. These findings indicate that CYP3A4 exerts opposite effects on the catalytic capacities of UGT1A7*1 and UGT1A7*4.

**Figure 3:**
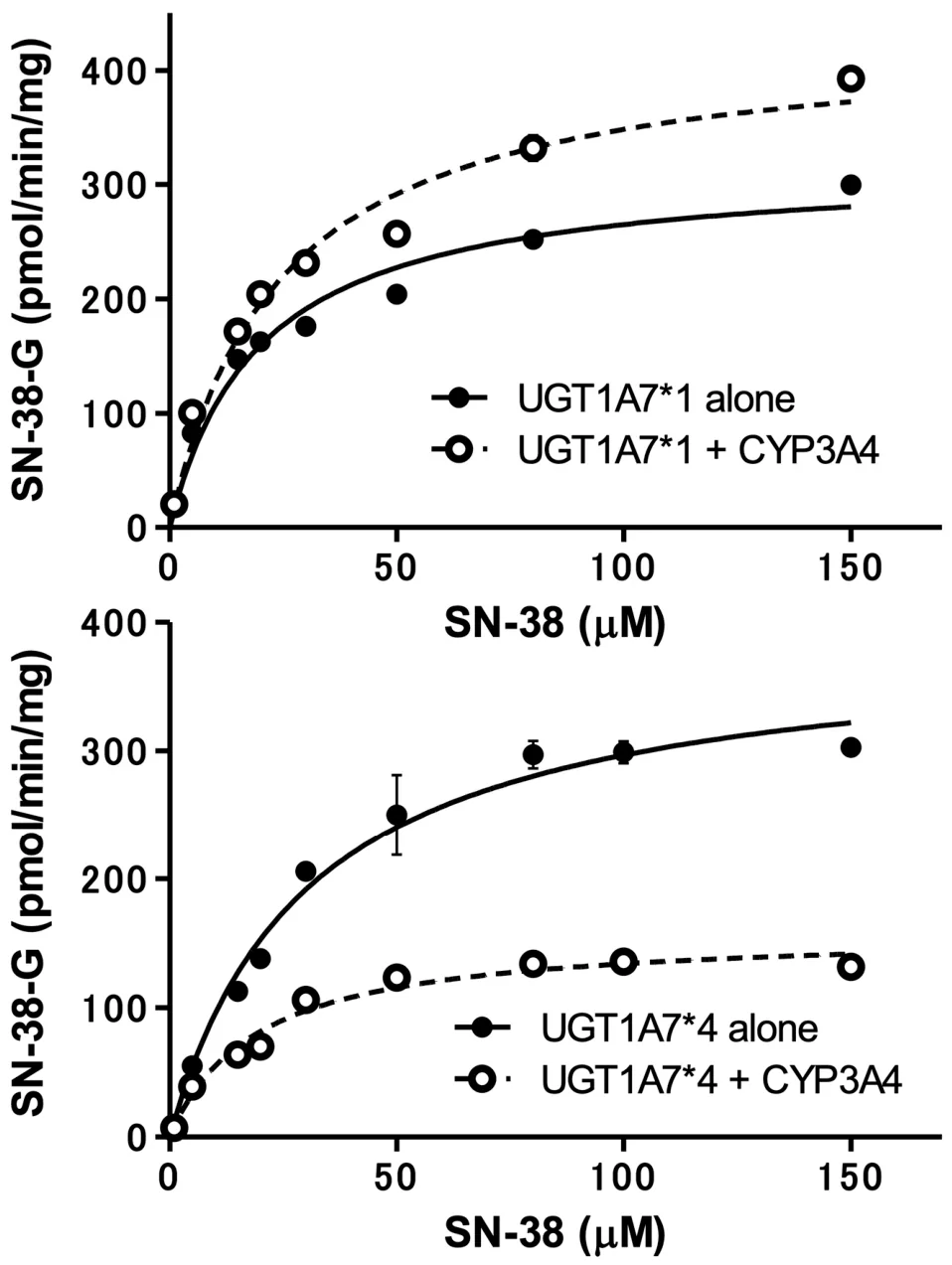
Differential effects of CYP3A4 on SN-38 glucuronidation catalyzed by UGT1A7 variants. (**Upper panel**) Michaelis–Menten plots for SN-38 glucuronidation catalyzed by UGT1A7*1 with and without CYP3A4 co-expression. (**Lower panel**) Michaelis–Menten plots for SN-38 glucuronidation catalyzed by UGT1A7*4 with and without CYP3A4 co-expression.

To determine whether this phenomenon was substrate-dependent, we then analyzed the glucuronidation of SN-38, the active metabolite of irinotecan and a physiologically relevant substrate of UGT1A7. Similarly to the results obtained with 4-MU, CYP3A4 enhanced UGT1A7*1-mediated SN-38 glucuronidation, whereas the same co-expression significantly suppressed the activity of UGT1A7*4 (Figure 3, Table 2). Although modest alterations in apparent substrate affinity were observed, the dominant effect of CYP3A4 was again a change in maximal catalytic activity.

**Figure 4:**
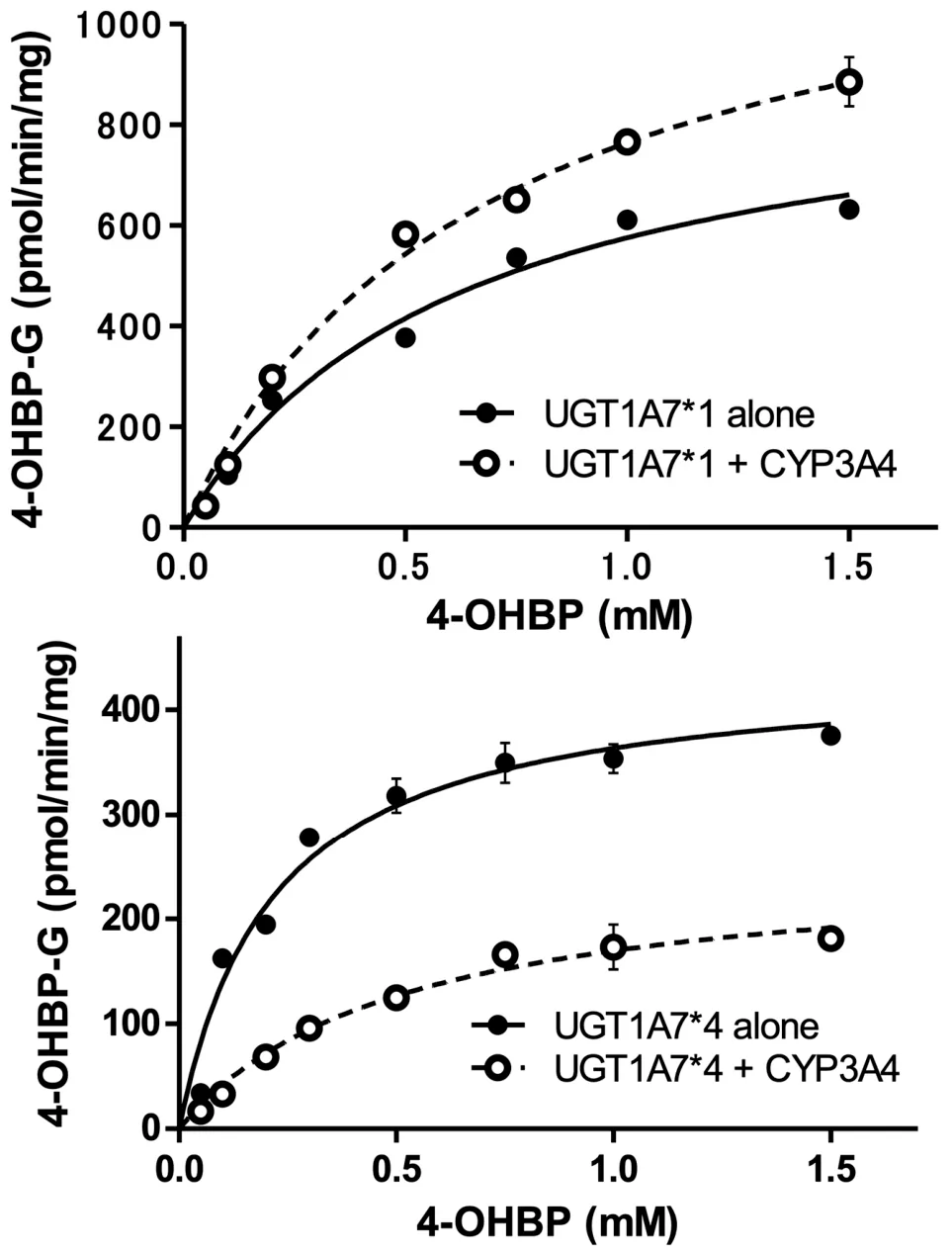
Differential effects of CYP3A4 on 4-hydroxybiphenyl (4-OHBP) glucuronidation catalyzed by UGT1A7 variants. (**Upper panel**) Michaelis–Menten plots for 4-OHBP glucuronidation catalyzed by UGT1A7*1 with and without CYP3A4 co-expression. (**Lower panel**) Michaelis–Menten plots for 4-OHBP glucuronidation catalyzed by UGT1A7*4 with and without CYP3A4 co-expression.

We further evaluated glucuronidation of 4-OHBP. To our best knowledge, this is the first report describing conjugation of 4-OHBP by UGT1A7. Co-expression of CYP3A4 increased the catalytic activity of UGT1A7*1 but decreased that of UGT1A7*4 (Figure 4, Table 3). In UGT1A7*4, both a reduction in *V*max and an increase in apparent *K*m were observed, resulting in a marked decrease in catalytic efficiency. Despite these kinetic differences, the overall direction of CYP3A4-dependent modulation remained consistent with those observed using 4-MU and SN-38.

**Figure 5:**
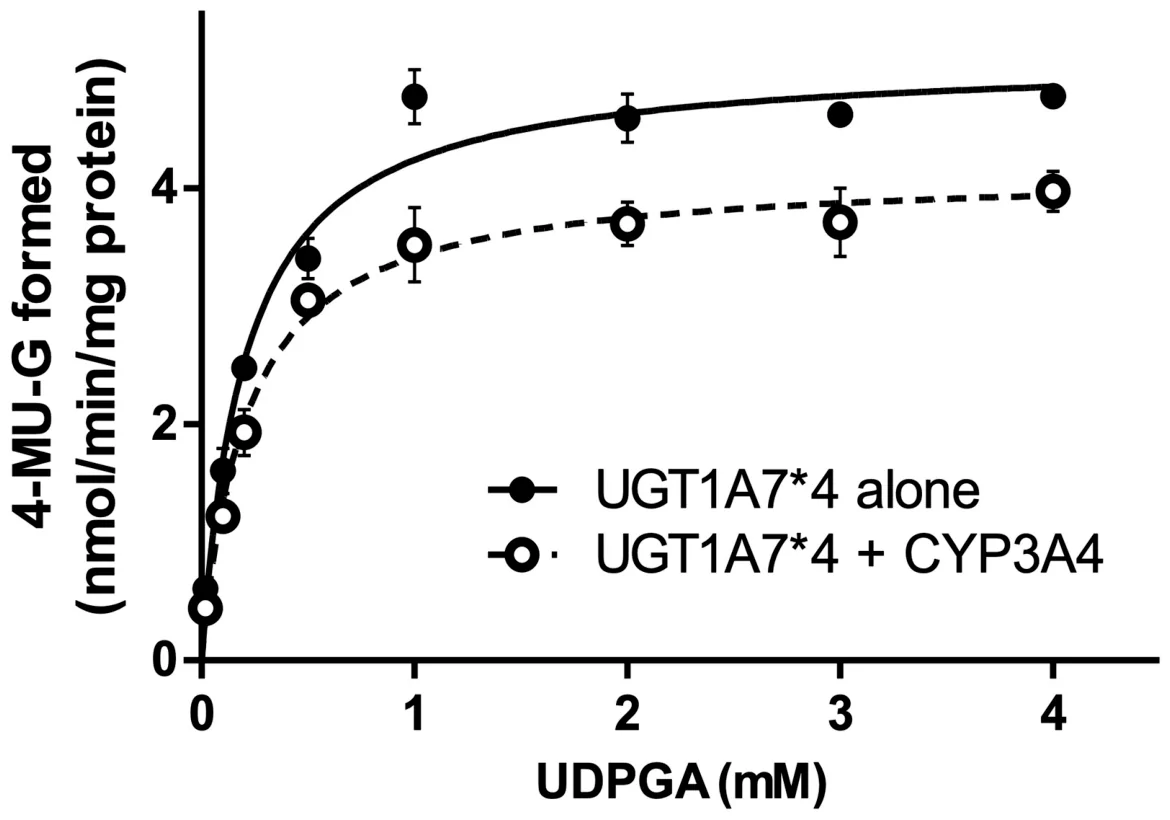
Comparison of 4-MU glucuronidation catalyzed by UGT1A7*4: UDPGA kinetics. Michaelis–Menten plots for 4-MU glucuronidation catalyzed by UGT1A7*4 in varying concentrations of UDPGA and fixing the concentration of 4-MU. See condition in Table 4.

As previous studies suggested that CYP3A4 may affect cofactor utilization by UGT1A7 variants, UDPGA-dependent kinetics were additionally examined for UGT1A7*4 [10]. CYP3A4 significantly reduced the *V*max of UGT1A7*4-catalyzed 4-MU glucuronidation, whereas the apparent affinity for UDPGA was largely unchanged (Figure 5, Table 4). These findings suggest that CYP3A4 mainly influences catalytic capacity rather than cofactor binding.

Taken together, these results demonstrate that CYP3A4 differentially regulates UGT1A7 variants depending on the amino acid present at residue 208. Whereas CYP3A4 consistently enhanced the catalytic activity of wild-type UGT1A7*1, it suppressed the activity of UGT1A7*4 carrying the W208R substitution. Importantly, this reversal of regulatory response was observed across three structurally distinct substrates, indicating that residue 208 is a critical determinant of the functional consequences of CYP3A4-mediated regulation of UGT1A7. These observations suggest that residue 208 is a critical functional determinant and may function as a molecular switch governing whether CYP3A4 stimulates or suppresses UGT1A7 catalytic function.

### 3.2. Functional Characterization of UGT1A7*4

UGT1A7*4 carrying the W208R substitution was successfully expressed in Sf9 cells using the baculovirus expression system. Immunoblot analysis demonstrated comparable expressions of UGT1A7*1 and UGT1A7*4, with both proteins migrating at approximately 50 kDa (Figure 1).

As mentioned above, it is possible that the W208R substitution could affect intrinsic catalytic properties of UGT1A7. Hence, enzyme kinetic analyses were performed using 4-MU and SN-38 as substrates. Both UGT1A7*1 and UGT1A7*4 exhibited Michaelis–Menten kinetics, and their apparent kinetic parameters were largely comparable (Table 1 and Table 2). Although kinetic parameters were generally similar, substrate-dependent differences were observed for 4-OHBP glucuronidation (Table 3).

These findings indicate that the W208R substitution does not markedly impair the intrinsic catalytic function of UGT1A7. Therefore, the opposite responses of UGT1A7*1 and UGT1A7*4 to CYP3A4 are unlikely to reflect differences in basal enzyme activity and instead suggest altered susceptibility to CYP3A4-mediated regulation.

### 3.3. Functional Importance of Residue 208 in UGT1A7 Activity

To further investigate the functional significance of residue 208 in UGT1A7, a mutant construct was generated in which Trp208 was replaced with alanine (W208A). This substitution was designed to evaluate whether the physicochemical properties of residue 208 contribute to the catalytic competence of UGT1A7.

**Figure 6:**
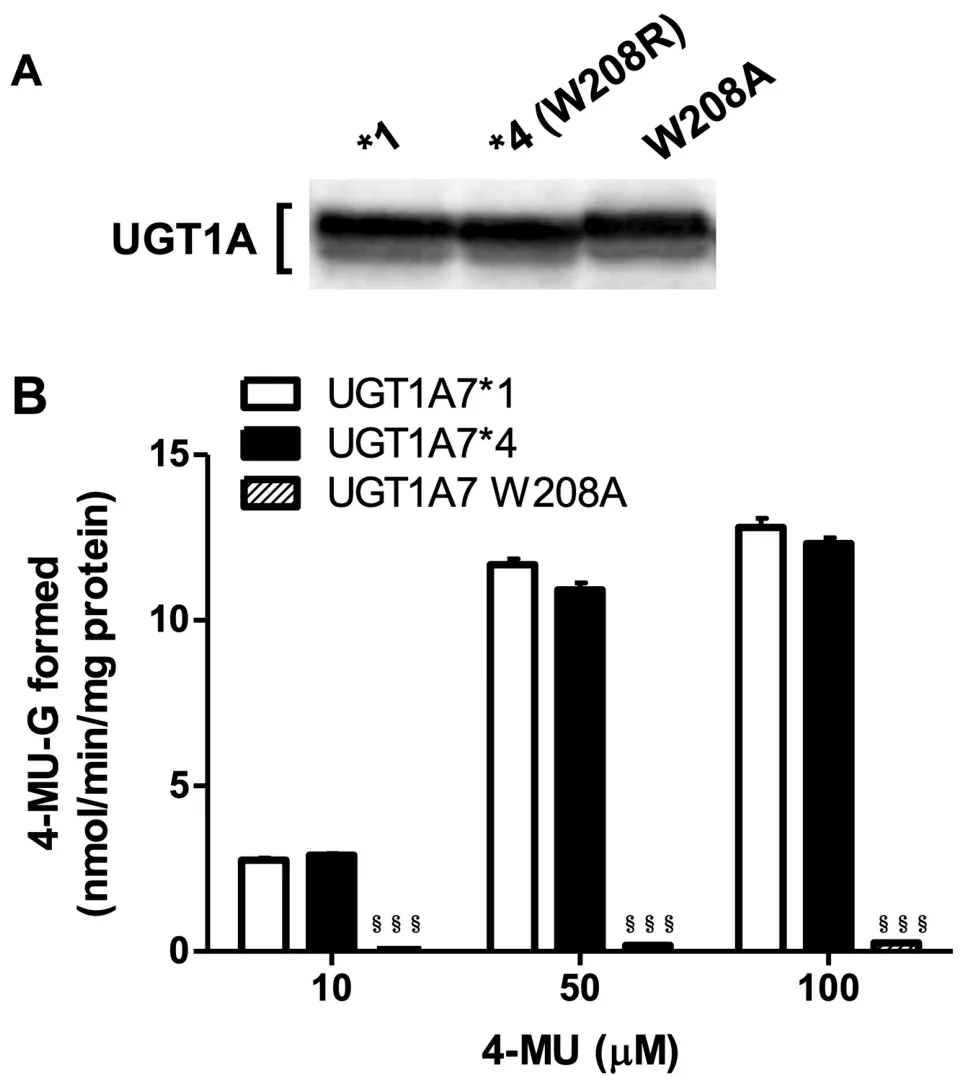
Functional importance of residue 208 in UGT1A7 activity. (**A**) Immunoblot analysis of UGT1A7*1, UGT1A7*4 (W208R), and the W208A mutant expressed in Sf9 microsomes. (**B**) Comparison of 4-MU glucuronidation activities of UGT1A7*1, UGT1A7*4, and W208A at the indicated substrate concentrations. Data represent means ± S.E. ^§§§^ *p* < 0.001 versus UGT1A7*1.

The W208A mutant was successfully expressed in Sf9 cells using the baculovirus expression system. Immunoblot analysis with an anti-UGT1A antibody detected an immunoreactive band corresponding to the recombinant protein (Figure 6A). Although the mutant protein was expressed, its electrophoretic mobility differed slightly from those of UGT1A7*1 and UGT1A7*4 (Figure 6A).

To evaluate the functional consequences of this substitution, glucuronidation activity toward 4-MU was measured at multiple substrate concentrations. As shown in Figure 6B, the W208A mutant retained detectable glucuronidation activity; however, its activity was markedly lower than those of both UGT1A7*1 and UGT1A7*4 across the concentration range examined. The decrease in activity was statistically significant at all substrate concentrations tested.

Interestingly, the naturally occurring W208R substitution present in UGT1A7*4 had little effect on basal catalytic activity (Section 3.2), whereas replacement of Trp208 with alanine resulted in a substantial loss of function. These findings indicate that residue 208 plays an important role in maintaining normal UGT1A7 activity. Furthermore, the preservation of catalytic activity in UGT1A7*4 suggests that the functional integrity of this position is not determined solely by the presence of tryptophan itself, but rather by the physicochemical characteristics of the residue occupying this position.

Although the mutant protein exhibited slightly altered electrophoretic mobility compared with UGT1A7*1 and UGT1A7*4, the molecular basis of this difference is presently unknown. Although alanine is smaller than either tryptophan or arginine, its substitution caused both a reduction in catalytic activity and a detectable change in migration behavior during SDS-PAGE.

Taken together, these findings demonstrate that residue 208 is a functionally important determinant of UGT1A7 activity. While the W208R substitution preserves basal catalytic function and primarily alters CYP3A4-dependent regulation, substitution with alanine substantially impairs enzymatic activity. These observations further support the conclusion that residue 208 plays a central role in the structure–function relationship of UGT1A7.

### 3.4. Residue 208 Does Not Affect Physical Association with CYP3A4

To determine whether the differential functional responses of UGT1A7 variants to CYP3A4 were attributable to altered protein–protein association, interactions between CYP3A4 and UGT1A7 variants were examined using a His-tag pull-down assay. UGT1A7*1, UGT1A7*4, and W208A were expressed as C-terminal HA-tagged proteins in COS-1 cells either alone or together with C-terminally His-tagged CYP3A4. Cell lysates were subjected to affinity purification using Ni^2+^-NTA agarose, and the resulting eluates were analyzed by immunoblotting.

**Figure 7:**
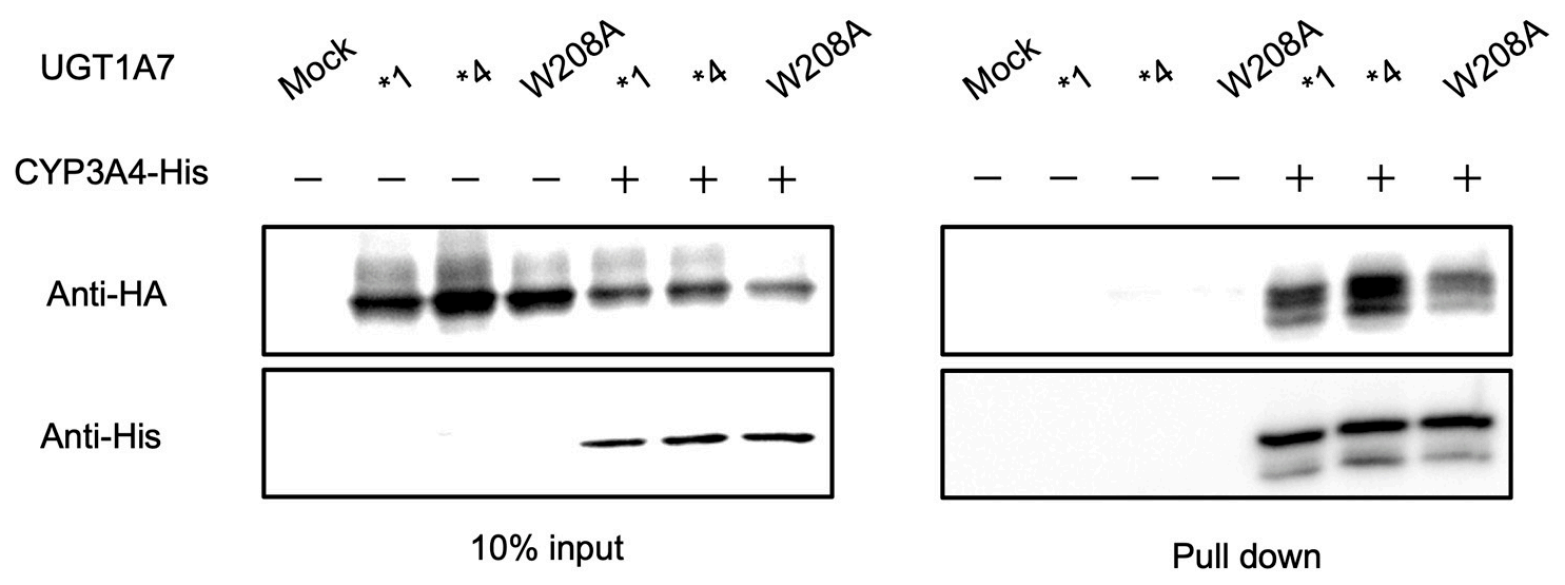
Physical association between CYP3A4 and UGT1A7 variants determined by His-tag pull-down assay. HA-tagged UGT1A7*1, UGT1A7*4, and W208A were transiently expressed in COS-1 cells with or without co-expression of C-terminally His-tagged CYP3A4 (CYP3A4-His). Cell lysates were subjected to pull-down using Ni^2+^-NTA agarose. **Upper panel**: Immunoblot detection of HA-tagged UGT1A7 proteins. **Lower panel**: Immunoblot detection of CYP3A4-His. Input fractions (10% input) and pull-down fractions are shown. UGT1A7*1, UGT1A7*4, and W208A were co-purified only in the presence of CYP3A4-His, indicating association of all three UGT1A7 proteins with CYP3A4.

Analysis of the input fractions confirmed the expression of all HA-tagged UGT1A7 proteins irrespective of CYP3A4 co-expression (Figure 7). In the pull-down fractions, no UGT1A7-derived immunoreactive bands were detected in the absence of CYP3A4-His. In contrast, clear UGT1A7 bands were observed when CYP3A4-His was co-expressed, indicating co-purification of UGT1A7 proteins with CYP3A4. This result was consistently observed for UGT1A7*1, UGT1A7*4, and the W208A mutant.

Importantly, neither the naturally occurring W208R substitution in UGT1A7*4 nor the experimental W208A substitution abolished the association between UGT1A7 and CYP3A4. Thus, despite exhibiting markedly different catalytic responses to CYP3A4, all three UGT1A7 proteins retained the ability to form complexes with CYP3A4.

These findings indicate that residue 208 is not essential for detectable association between UGT1A7 and CYP3A4. Instead, the opposite regulatory effects observed for UGT1A7*1 and UGT1A7*4 are more likely to result from alterations in the functional consequences of complex formation rather than from changes in protein–protein binding itself.

### 3.5. CYP3A4 Interacts with UGT1A7 in Living Cells

To investigate whether the association between CYP3A4 and UGT1A7 occurs in living cells, FRET analysis was performed using fluorescent fusion proteins. UGT1A7*1 and UGT1A7*4 were fused to AcGFP1, whereas CYP3A4 was fused to mCherry. As a control, CNX, an endoplasmic reticulum membrane protein with a membrane topology similar to that of UGT1A7, was fused to AcGFP1 and co-expressed with CYP3A4-mCherry in COS-1 cells.

Forty-eight hours after transfection, live cells were analyzed by confocal laser-scanning microscopy. Distinct fluorescence signals derived from AcGFP1 and mCherry were detected in all transfected cells, confirming successful expression of the fusion proteins. FRET signals were then evaluated using a sensitized emission approach after correction for spectral bleed-through.

**Figure 8:**
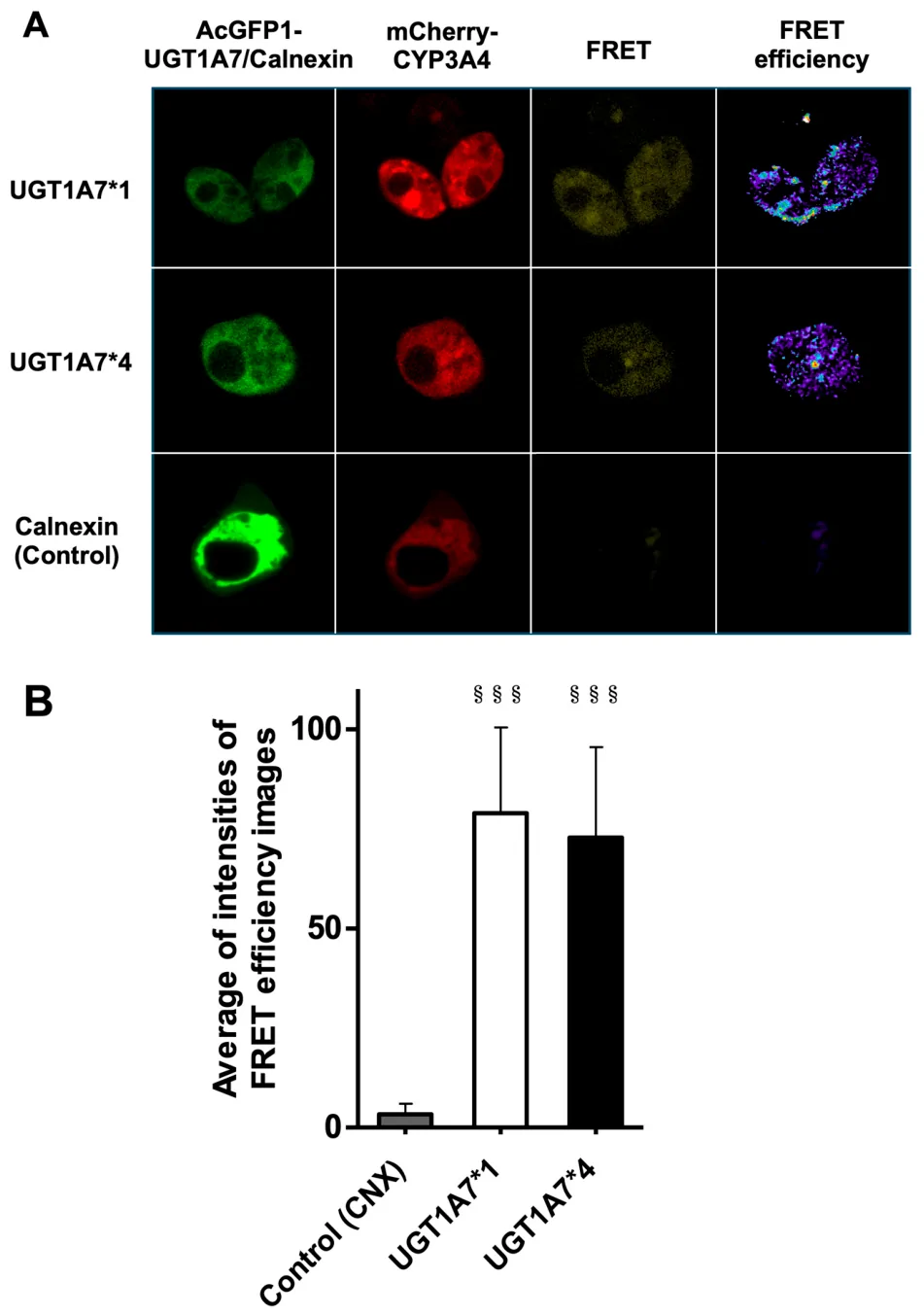
FRET analysis demonstrates interaction between CYP3A4 and UGT1A7 in living cells. (**A**) Representative confocal images of COS-1 cells co-expressing UGT1A7*1-AcGFP1, UGT1A7*4-AcGFP1, or calnexin (CNX)-AcGFP1 together with CYP3A4-mCherry. AcGFP1, mCherry, merged, and FRET images are shown. (**B**) Quantification of FRET efficiency. FRET efficiencies were calculated using sensitized emission analysis. Ten cells were analyzed for each construct. Values represent means ± S.E. ^§§§^ *p* < 0.001 versus CNX control.

As shown in Figure 8A, strong FRET signals were observed in cells co-expressing UGT1A7*1-AcGFP1 and CYP3A4-mCherry. Comparable signals were also detected in cells co-expressing UGT1A7*4-AcGFP1 and CYP3A4-mCherry. In contrast, substantially weaker FRET signals were observed in control cells expressing CNX-AcGFP1 together with CYP3A4-mCherry.

Quantitative analysis of FRET efficiency demonstrated that both UGT1A7*1 and UGT1A7*4 exhibited significantly higher FRET efficiencies than the CNX control (Figure 8B). No statistically significant difference was detected between UGT1A7*1 and UGT1A7*4.

These findings indicate that CYP3A4 physically associates with UGT1A7 in living cells and further demonstrate that the W208R substitution does not disrupt this interaction. Although CYP3A4 enhanced the catalytic activity of UGT1A7*1 while suppressing that of UGT1A7*4, both variants exhibited comparable FRET efficiencies, suggesting that the opposite functional responses are more likely to result from alterations in the functional consequences of complex formation. The present pull-down assay demonstrated detectable association but did not permit a quantitative comparison of binding strength.

Together with the pull-down results presented above, the FRET analysis provides complementary biochemical and cellular evidence that UGT1A7 and CYP3A4 form protein–protein complexes. These observations support the conclusion that residue 208 regulates the functional consequences of CYP3A4-UGT1A7 association rather than the formation of the interaction itself.

## 4. Discussion

### 4.1. Residue 208 Determines CYP3A4 Responsiveness Rather than Basal UGT1A7 Activity

Our previous study demonstrated that CYP3A4 enhanced the catalytic activities of UGT1A7*1 and UGT1A7*2, whereas it suppressed the activity of UGT1A7*3 [10]. Because UGT1A7*3 differs from UGT1A7*2 by a single amino acid substitution at residue 208 (W208R), residue 208 was proposed as a critical determinant of CYP3A4-dependent regulation. However, direct experimental evidence supporting this hypothesis was lacking because UGT1A7*3 contains multiple amino acid substitutions. The present study addressed this unresolved question through analysis of UGT1A7*4, a naturally occurring variant carrying only the W208R substitution.

UGT1A7*4 exhibited basal catalytic activity comparable with that of wild-type UGT1A7*1, indicating that the W208R substitution itself does not markedly impair catalytic function. In contrast, co-expression of CYP3A4 reversed the direction of regulation, converting CYP3A4-mediated activation observed with UGT1A7*1 into suppression in UGT1A7*4. Importantly, this reversal was consistently observed with three structurally distinct substrates, namely, 4-MU, SN-38, and 4-OHBP. These observations establish that residue 208 is a critical determinant of CYP3A4 responsiveness rather than a major determinant of basal catalytic activity.

### 4.2. Residue 208 Is Not Required for Detectable Association Between CYP3A4 and UGT1A7

One possible explanation for the differential effects of CYP3A4 on UGT1A7 variants would be that the W208R substitution alters the ability of UGT1A7 to interact physically with CYP3A4. However, the results obtained in the present study do not support this hypothesis. His-tag pull-down assays demonstrated that UGT1A7*1, UGT1A7*4, and the W208A mutant all retained the ability to associate with CYP3A4. Furthermore, FRET analysis revealed efficient interactions between CYP3A4 and both UGT1A7*1 and UGT1A7*4 in living cells.

These findings indicate that the W208R substitution does not abolish protein–protein association. Instead, detectable association is maintained irrespective of the amino acid present at position 208. Consequently, the opposite functional responses observed for UGT1A7*1 and UGT1A7*4 cannot be attributed simply to gain or loss of binding between CYP3A4 and UGT1A7.

This distinction is important because many previous studies of CYP-UGT interactions have focused primarily on the existence of physical association. The present findings suggest that the functional consequences of an interaction may be regulated independently of interaction formation itself.

### 4.3. Residue 208 May Function as a Putative Molecular Switch Governing the Functional Consequences of CYP3A4-UGT1A7 Association

The most notable observation in the present study is that the physical association between CYP3A4 and UGT1A7 was preserved despite complete reversal of the regulatory response. This finding suggests that residue 208 does not function as a binding determinant but rather as a regulator of the functional consequences of the CYP3A4-UGT1A7 association.

Further support for this interpretation was obtained from the W208A mutant. Although the mutant retained the ability to associate with CYP3A4, its catalytic activity was substantially impaired. These observations imply that residue 208 contributes to maintaining the structural and functional integrity of UGT1A7. The replacement of Trp with Arg preserved basal activity but altered the response to CYP3A4, whereas replacement with Ala markedly impaired catalytic function. Together, these findings suggest that subtle changes in the physicochemical properties of residue 208 may influence conformational responses induced by CYP3A4 association.

Accordingly, residue 208 may function as a molecular switch that determines whether CYP3A4-mediated association results in activation or suppression of UGT1A7 activity. This concept extends earlier observations made with UGT1A7 polymorphic variants and provides direct experimental evidence for the critical role of a single amino acid residue in determining the outcome of CYP-UGT functional interaction.

### 4.4. Implications for Current Models of CYP-UGT Interactions

Accumulating evidence indicates that P450 enzymes and UGTs form a functionally integrated network within the endoplasmic reticulum membrane. Previous studies have demonstrated interactions between CYP3A4 and multiple UGT isoforms, including UGT2B7, UGT1A1, UGT1A9, and UGT1A7, with consequences for both CYP and UGT activities. Protein–protein interactions between cytochrome P450 enzymes and UGTs have been investigated for more than two decades. Our group first reported the existence of CYP–UGT interactions in 2000 [6]. Subsequently we demonstrated that CYP3A4 modulates UGT catalytic activity through protein–protein association [8]. Around the same period, King and colleagues provided additional biochemical evidence supporting physical association between CYP3A4 and UGT1A1, 1A6 and 2B7 [7]. While studies of CYP-CYP interactions by Backes and colleagues established the broader concept that interactions among drug-metabolizing enzymes can alter catalytic function [13], early demonstrations of functional modulation in CYP-UGT systems were largely derived from studies conducted by our group [8,9,28].

Proteomics approaches were conducted to find candidate proteins interacting with CYP3A4 and UGT2B7 [29,30]. More recent studies employing interactome-scale approaches and chemical crosslinking-mass spectrometry have further supported the existence of extensive interactions between P450 enzymes and UGTs in human liver microsomes [14,31].

Within this evolving framework, the present study provides two important advances. First, FRET analysis provides direct evidence that CYP3A4 and UGT1A7 interact in living cells. Previous demonstrations of P450-UGT interactions primarily relied on biochemical approaches performed after cell disruption, including co-immunoprecipitation, pull-down assays, affinity chromatography, and crosslinking experiments [6,7,9,11,12]. The present study therefore extends previous findings into a cellular context.

Second, our results demonstrate that the presence of protein–protein association alone is insufficient to predict functional outcomes. Instead, the consequences of CYP3A4-UGT1A7 interaction depend on the molecular properties of the interacting proteins. This concept is consistent with emerging views that drug-metabolizing enzymes function as components of highly integrated multiprotein networks rather than as independent catalytic entities.

Previous studies have demonstrated that UGT proteins are capable of forming homo- and hetero-oligomeric complexes. In particular, Operaña and Tukey employed co-immunoprecipitation and fluorescence resonance energy transfer (FRET) analyses to demonstrate direct interactions among UGT isoforms, providing important evidence that UGTs function within higher-order protein assemblies [32]. These studies established the utility of FRET for investigating membrane-associated UGT interactions.

In contrast, evidence for direct CYP-UGT interactions in living cells has remained limited. Although biochemical approaches, including affinity chromatography, co-immunoprecipitation, pull-down assays, and crosslinking experiments, have supported physical association between CYPs and UGTs [6,7,9,11,12], visualization of these interactions in living cells has not been reported to our knowledge. Therefore, the present study extends previous work by demonstrating the CYP3A4-UGT1A7 interaction using FRET analysis in living cells.

Importantly, our results further show that preservation of detectable association does not necessarily predict functional outcome. UGT1A7*1 and UGT1A7*4 exhibited comparable interactions with CYP3A4 in both pull-down and FRET analyses yet displayed opposite catalytic responses to CYP3A4 co-expression. These observations indicate that the molecular determinants governing the consequences of protein–protein interaction may be distinct from those governing interaction formation itself.

### 4.5. Limitations and Future Perspectives

Several limitations should be acknowledged. Although the present study establishes the importance of residue 208 in determining the functional outcome of the CYP3A4-UGT1A7 interaction, the structural mechanism responsible for this phenomenon remains unresolved. The direct interaction interface between CYP3A4 and UGT1A7 has not yet been identified, and the conformational changes induced by residue 208 substitutions remain unknown.

Future studies employing structural approaches, including molecular modeling, crosslinking-based interaction mapping, and emerging high-resolution structural methods, may provide important insight into how residue 208 influences communication between CYP3A4 and UGT1A7. Such investigations will contribute to a more complete understanding of the molecular basis of CYP-UGT interactions and their contribution to interindividual variation in drug metabolism.

Our findings suggest that residue 208 may function as a molecular switch that determines the functional outcome, rather than the physical occurrence, of CYP3A4-UGT1A7 association.

## 5. Conclusions

In the present study, we investigated the role of residue 208 in the functional interaction between CYP3A4 and UGT1A7. Analysis of the naturally occurring variant UGT1A7*4 demonstrated that substitution of Trp208 with Arg had little effect on the intrinsic catalytic activity of UGT1A7 but profoundly altered its response to CYP3A4-mediated regulation. Whereas CYP3A4 enhanced the activity of wild-type UGT1A7*1, it consistently suppressed the activity of UGT1A7*4, indicating that residue 208 is a critical determinant of CYP3A4 responsiveness.

Pull-down assays demonstrated that UGT1A7*1, UGT1A7*4, and the W208A mutant all retained the ability to associate with CYP3A4. Furthermore, FRET analysis provided direct evidence that CYP3A4 and UGT1A7 interact in living cells. Together, these findings indicate that residue 208 does not determine the formation of CYP3A4-UGT1A7 complexes but rather influences the functional consequences of their association.

The present study therefore identifies that residue 208 may function as a determinant, and potentially as a molecular switch, that governs whether CYP3A4 exerts positive or negative regulation on UGT1A7 activity. More broadly, our findings support the concept that the biological consequences of protein–protein interactions among drug-metabolizing enzymes are not determined solely by interaction formation but also by the structural characteristics of the interacting proteins. These results provide new insight into the molecular basis of CYP-UGT interactions and contribute to our understanding of post-translational mechanisms underlying interindividual variation in drug metabolism.

## Figures and Tables

**Table 1:** Apparent kinetic parameters for 4-MU glucuronide formation catalyzed by microsomes from Sf9 cells expressing UGT1A7*1/*4 alone or UGT1A7*1/*4 + CYP3A4: kinetics as a function of 4-MU concentration.

	Km (μM)	*V*max (nmol/min/mg Protein)	*V*max/*K*m
*1	22.5 ± 3.0	6.63 ± 0.24	0.29
*1 + CYP3A4	28.3 ± 2.8	11.0 ± 0.31 ^§§§^	0.39
*4	18.9 ± 2.2	6.48 ± 0.19	0.34
*4 + CYP3A4	13.7 ± 1.9	3.98 ± 0.12 ^§§§^	0.29

Data were fitted to a Michaelis–Menten equation. Results are the estimated value ± S.E. UGT activity was determined with substrate concentrations ranging from 1 to 300 μM. UDPGA concentration was fixed at 2 mM. ^§§§^ Significantly different from single expression (*p* < 0.0001).

**Table 2:** Apparent kinetic parameters for SN-38 glucuronide formation catalyzed by microsomes from Sf9 cells expressing UGT1A7*1/*4 alone or UGT1A7*1/*4 + CYP3A4: kinetics as a function of SN-38 concentration.

	*K*m (μM)	*V*max (nmol/min/mg Protein)	*V*max/*K*m
*1	19.9 ± 2.2	318 ± 11	16.0
*1 + CYP3A4	24.1 ± 2.5	432 ± 15 ^§§§^	17.9
*4	30.7 ± 3.0	388 ± 13	12.6
*4 + CYP3A4	19.0 ± 2.0	160 ± 5 ^§§§^	8.42

Data were fitted to a Michaelis–Menten equation. Results are the estimated value ± S.E. UGT activity was determined with substrate concentrations ranging from 1 to 150 μM. UDPGA concentration was fixed at 2 mM. ^§§§^ Significantly different from single expression (*p* < 0.0001).

**Table 3:** Apparent kinetic parameters for 4-OHBP glucuronide formation catalyzed by microsomes from Sf9 cells expressing UGT1A7*1/*4 alone or UGT1A7*1/*4 + CYP3A4: kinetics as a function of 4-OHBP concentration.

	*K*m (mM)	*V*max (nmol/min/mg Protein)	*V*max/*K*m
*1	0.63 ± 0.08	934 ± 52	1480
*1 + CYP3A4	0.72 ± 0.08	1316 ± 64 ^§§§^	1830
*4	0.22 ± 0.03	442 ± 17	2010
*4 + CYP3A4	0.52 ± 0.07 ^§§§^	257 ± 14 ^§§§^	494

Data were fitted to a Michaelis–Menten equation. Results are the estimated value ± S.E. UGT activity was determined with substrate concentrations ranging from 0.05 to 1.5 mM. UDPGA concentration was fixed at 2 mM. ^§§§^ Significantly different from single expression (*p* < 0.0001).

**Table 4:** Apparent kinetic parameters for 4-MU glucuronide formation catalyzed by microsomes from Sf9 cells expressing UGT1A7*4 alone or UGT1A7*4 + CYP3A4: kinetics as a function of UDPGA concentration.

	*K*m (mM)	*V*max (nmol/min/mg Protein)	*V*max/*K*m
*4	0.22 ± 0.03	5.13 ± 0.12	23.3
*4 + CYP3A4	0.23 ± 0.02	4.18 ± 0.10 ^§§§^	18.2

Data were fitted to a Michaelis–Menten equation. Results are the estimated value ± S.E. UGT activity was determined using 8 cofactor concentrations ranging from 0.05 to 4 mM. The 4-MU concentration was fixed at 100 μM. ^§§§^ Significantly different from single expression (*p* < 0.0001).

## Data Availability

All data are presented in this manuscript and in the Supplementary Materials.

## Supplementary Materials

The following supporting information can be downloaded at https://www.mdpi.com/article/10.3390/biology15201895/s1, Table S1: Primers used in this study; Methods S1. PCR conditions used for site-directed mutagenesis; construction of UGT1A7*4 (W208R); Table S2: PCR conditions used for site-directed mutagenesis; Table S3: Multiplicity of infection (MOI) conditions used for expression of UGT1A7 variants and CYP3A4; Table S4: Detailed chromatographic conditions used for quantification of UGT reaction products.

## Abbreviations

The following abbreviations are used in this manuscript:

CYP3A4	Cytochrome P450 3A4
UGT	UDP-glucuronosyltransferase
FRET	Fluorescence resonance energy transfer
4-MU	4-Methylumbelliferone
4-OHBP	4-Hydroxybiphenyl
MOIs	Multiplicities of infection
CNX	Calnexin
UDPGA	UDP-glucuronic acid
HA	Hemagglutinin
