# Supplementary material for: Residue 208 of UGT1A7 Determines the Functional Consequences of CYP3A4-UGT1A7 Association in Living Cells
Source: Biology (Basel). 2026 Oct 21;15(20):1895. doi: 10.3390/biology15201895 (PMC13701601; doi:10.3390/biology15201895)
Supplement: Supplementary file 1 [file biology-15-01895-s001.zip › biology-4624119-supplementary.pdf]

Residue 208 of UGT1A7 Determines the Functional Consequences of CYP3A4-UGT1A7 Association in Living Cells

Supplementary

Supplementary Table S1. Primers Used in This Study

Site-Directed Mutagenesis of UGT1A7 Variants

Generation of UGT1A7*4 (W208R)

Primer	Sequence (5′→3′)
UGT1A7-W208R-F	CTTTCAAGGAGAGAGTACGGAACCACATCATGCAC
UGT1A7-W208R-R	GTGCATGATGTGGTTCCGTACTCTCTCCTTGAAAG

The mutated nucleotides introducing the W208R substitution are indicated in bold in the original primer design.

Generation of UGT1A7 W208A

Primer	Sequence (5′→3′)
UGT1A7-W208A-F	GACTTTCAAGGAGAGAGTAGCGAACCACATCATGCACTTG
UGT1A7-W208A-R	CAAGTGCATGATGTGGTTCGCTACTCTCTCCCTTGAAAGTC

The mutated nucleotides introducing the W208A substitution are indicated in bold in the original primer design.

Primers Used for DNA Sequencing

Verification of UGT1A7*4 Constructs

Primer	Sequence (5′→3′)

pFastBac-F	TAAAAAAACCTATAAATATTCCGG
hUGT1A1-R	CTCCATGCGCTTTGCATTGT
pFastBac-R	TGAAACATAAAATGAATGCAATTG

Supplementary Table S1. Primers used for site-directed mutagenesis and sequence verification of UGT1A7 constructs. Primers were used to generate the UGT1A7*4 (W208R) variant and the W208A mutant by PCR-based site-directed mutagenesis. Additional primers were used for sequence verification of recombinant constructs.

Supplementary Methods S1. PCR Conditions Used for Site-Directed Mutagenesis

Construction of UGT1A7*4 (W208R)

UGT1A7*_4 cDNA was generated from UGT1A7_*1 using site-directed mutagenesis. PCR amplification was performed with PfuTurbo DNA polymerase using the following reaction mixture and cycling conditions.

PCR reaction mixture

- 10× Pfu buffer: 5 µL
- Forward primer (5 µM): 3 µL
- Reverse primer (5 µM): 3 µL
- dNTP mixture (2.5 mM each): 1 µL
- PfuTurbo DNA polymerase: 1 µL
- Template DNA (pFastBac1-UGT1A7*1): 1 µL (50 ng/µL)
- Sterile water: 36 µL

Total volume: 50 µL

PCR cycling conditions

Step	Temperature	Time	Cycles
Initial denaturation	94°C	2 min	1
Denaturation	98°C	10 s	30
Annealing	60°C	30 s	
Extension	68°C	3 min 30 s	
Hold	4°C	∞	1

Supplementary Table S2. PCR Conditions Used for Site-Directed Mutagenesis

UGT1A7*4 (W208R)

PCR was performed using PfuTurbo DNA polymerase with UGT1A7*1 as the template. Amplification conditions were as follows.

Step	Temperature	Time	Cycles
Initial denaturation	94°C	2 min	1
Denaturation	98°C	10 s	30
Annealing	60°C	30 s	
Extension	68°C	3 min 30 s	
Final hold	4°C	∞	1

PCR products were treated with *Dpn*I and subsequently used for bacterial transformation.

UGT1A7 W208A

Site-directed mutagenesis was performed using the same PCR mixture as described for generation of UGT1A7*4. Amplification conditions were optimized according to the melting temperatures of the mutation-specific primers.

Step	Temperature	Time	Cycles
Initial denaturation	94°C	2 min	1

| Denaturation | 98°C | 10 s | 30 | | Annealing | Optimized according to primer Tm | 30 s | | | Extension | 68°C | 3 min 30 s | | | Final hold | 4°C | ∞ | 1 |

PCR Reaction Mixture

The following reaction mixture was used for site-directed mutagenesis.

Component	Volume
10× Pfu buffer	5 µL
Forward primer (5 µM)	3 µL
Reverse primer (5 µM)	3 µL
dNTP mixture (2.5 mM each)	1 µL
PfuTurbo DNA polymerase	1 µL
Template DNA	1 µL
Sterile water	36 µL
**Total volume**	**50 µL**

Table Legend

Supplementary Table S2. PCR conditions used for construction of UGT1A7 mutants. Site-directed mutagenesis was performed using UGT1A7*1 cDNA as a template. UGT1A7*4 carrying the W208R substitution and the W208A mutant were generated by PCR-based mutagenesis followed by *Dpn*I digestion and sequence verification.

Supplementary Table S3. Multiplicity of Infection (MOI) Conditions Used for Expression of UGT1A7 Variants and CYP3A4

Supplementary Table S3. Baculovirus infection conditions used for expression of UGT1A7 variants and CYP3A4 in Sf9 cells. Infection conditions were optimized to obtain comparable expression levels of recombinant proteins for enzymatic and protein-protein interaction analyses. Cells were harvested approximately 60–65 h after infection. For CYP3A4-containing cultures, hemin was added 24 h after infection to a final concentration of 1 μg/mL.

Expression System	Recombinant Virus	MOI
UGT1A7*1 single expression	UGT1A7*1	0.0001
UGT1A7*1 + CYP3A4 co-expression	UGT1A7*1	0.0002
	CYP3A4	0.001
UGT1A7*4 single expression	UGT1A7*4	0.00005
UGT1A7*4 + CYP3A4 co-expression	UGT1A7*4	0.0001
	CYP3A4	0.001
W208A single expression	W208A	0.016

（Table Footnote）

MOI, multiplicity of infection (plaque-forming units per cell). Infection conditions were selected to yield comparable UGT1A7 expression levels as assessed by immunoblot analysis. Hemin was added to cultures containing CYP3A4 to promote formation of catalytically active holo-CYP3A4.

Supplementary Table S4. Detailed Chromatographic Conditions Used for Quantification of UGT Reaction Products

Supplementary Table S4. Detailed chromatographic conditions used for determination of 4-MU glucuronide, SN-38 glucuronide, and 4-OHBP glucuronide.

A. 4-MU Glucuronide

The HITACHI Chromaster series shown below was used.

5110 pump, 5210 Autosampler, 5440 FL Detector, 5420 UV-VIS Detector, 5310 Column Oven

Parameter	Condition
Column	Nova Pak C18(4 μm) cartridge (8×10) packed into an RCM module (8×10) was connected to a guard column (Nova Pak® 4 mm 60 Å C18, Guard Pak™, HPLC Precolumn Inserts)
Mobile phase	Solvent A: Solvent B = 80 : 20 (1–10 min) → 50 : 50 (10–13 min) → 80 : 20 (13–20 min)<br><br>Solvent A: 20 mM sodium phosphate buffer (pH 2.2)<br><br>Solvent B: CH_3CN
Ratio	Programmed as above
Flow rate	0.8 mL/min
Column temperature	37°C
Detection	Fluorescence
Excitation wavelength	315 nm
Emission wavelength	375 nm
Injection volume	20 μL

B. SN-38 Glucuronide

SN-38 glucuronide was quantified according to the HPLC method of Hanioka et al. with minor modifications.

Parameter	Condition
Column	Nova Pak C18(4 μm) cartridge (8×10) packed into an RCM module (8×10) was connected to a guard column (Nova Pak® 4 mm 60 Å C18, Guard Pak™, HPLC Precolumn Inserts)
Mobile phase	Solvent A: Solvent B = 95 : 5 → 50 : 50 (0–18 min) / 50 : 50 → 10 : 90 (18–23 min) / 10 : 90 → 95 : 5 (23–30 min) / 95 : 5 (30–35 min)<br><br>Solvent A: 20 mM sodium phosphate buffer (pH 2.2)<br><br>Solvent B: CH_3CN
Ratio	Programmed as above
Flow rate	0.8 mL/min
Column temperature	37°C
Detection	Fluorescence
Excitation wavelength	370 nm
Emission wavelength	425 nm
Injection volume	20 μL

Reference:
Hanioka, N.; Jinno, H.; Nishimura, T.; Ando, M.; Ozawa, S.; Sawada, J. *Biomed. Chromatogr.* **2001**, 15, 328–333.

C. 4-OHBP Glucuronide

4-OHBP glucuronide standard was prepared according to the procedure of Oguri et al. and used for chromatographic identification.

Parameter	Condition
Column	Nova Pak C18(4 μm) cartridge (8×10) packed into an RCM module (8×10) was connected to a guard column (Nova Pak® 4 mm 60 Å C18, Guard Pak™, HPLC Precolumn Inserts)
Mobile phase	Solvent A: Solvent B = 5 : 95 (1–9.9 min) → 95 : 5 (10–19.9 min) → 5 : 95 (20–30 min)<br><br>Solvent A: 20 mM sodium phosphate buffer (pH 2.2)<br><br>Solvent B: CH_3CN
Ratio	Programmed as above
Flow rate	0.8 mL/min
Column temperature	37°C
Detection	Fluorescence
Excitation wavelength	269 nm
Emission wavelength	323 nm
Injection volume	20 μL

Footnote

Chromatographic conditions represent those used for routine quantification of glucuronide products throughout this study. SN-38 glucuronide analysis was performed essentially according to the method of Hanioka et al. (2001). 4-OHBP glucuronide standard was prepared using the procedure described by Oguri et al. (1996).
